# Simulation and Experimental Study of a Novel Negative-Pressure Flapper–Nozzle Mechanism

**DOI:** 10.3390/mi15010055

**Published:** 2023-12-26

**Authors:** Aixiang Ma, Heruizhi Xiao, Xihao Yan, Xianghao Kong, Feng Rong, Lu Zhang, Sihai Zhao

**Affiliations:** School of Mechanical and Electrical Engineering, China University of Mining & Technology, Beijing 100083, China

**Keywords:** adjusting valve positioner, flapper–nozzle, negative-pressure nozzle, CFD, compressible fluid

## Abstract

As the manufacturing industry evolves, the significance of control valve positioners in chemical production escalates. The flapper–nozzle system, the heart of control valve positioners, directly influences the linearity of system control. Presently, studies on the flapper–nozzle system primarily focus on dynamic system modeling and computational fluid dynamics simulations. However, traditional flapper–nozzle mechanisms often fail to achieve linear control objectives. This paper proposes a novel negative-pressure nozzle structure to tackle this issue, combining computational fluid dynamics and experimental methods, and considering gas compressibility during high-speed flow. Both simulation and experimental results suggest that the new structure improves the supply air pressure and broadens the linear pressure output range of the system, showing significant potential for practical applications.

## 1. Introduction

Valve positioners are essential control instruments in pneumatic control valve systems, comprising an electro-pneumatic converter, a displacement amplifier, a pressure amplification device, and other components. They receive and process the pressure signal from the air source input and feedback signals from the feedback mechanism, thus modifying the output pressure signal to drive the actuator’s motion, which alters the opening between the valve core and the valve. Based on the principles of the electro-pneumatic converter, valve positioners can be categorized into flapper–nozzle-type valve positioners and piezoelectric ceramic-type valve positioners. Currently, flapper–nozzle–type valve positioners are more prevalent in industrial applications due to their lower requirements for air source cleanliness, as well as their durability, compact size, quick response times, sensitivity to adjustments, and wide bandwidth [1].

The flapper–nozzle, the core component of this type of valve positioner, directly impacts the control precision of the valve positioner. Flapper–nozzle valves are extensively used in hydraulic and pneumatic systems, particularly for control and measurement purposes [2]. However, in valve position control systems, flapper–nozzle structures suffer from a limited linear operating range. Owing to cost limitations, the flapper–nozzle mechanism is typically driven by electromagnetic solenoids and traditional pneumatic flapper–nozzle systems have a limited linear range, making it challenging to ensure accurate operation throughout the entire range [3,4]. The limited linear range of pneumatic flapper–nozzle systems restricts their positioning accuracy and makes them unsuitable for certain high-precision control valve positioning applications. To enhance the performance of flapper–nozzle mechanisms, new solutions, and technical means need to be proposed. Numerous scholars have undertaken mathematical and nonlinear dynamic modeling and research, ranging from linearized algebraic equations to nonlinear dynamic model control systems [5,6,7,8,9]. Key structural parameters of nozzles significantly influence the flow characteristics of the internal energy transfer medium and are a focal point of fundamental theoretical research and design development. Several scholars have employed numerical simulation methods to study the flow characteristics of flapper–nozzle valves, including single flapper–nozzle, double flapper–nozzle, and jet pipe electro-hydraulic servo valves. They have investigated the internal flow field distribution [10,11], hydrodynamic forces on the valve core [12], temperature [13], vibration, and cavitation [14,15,16], among others. While there is a substantial body of research on the structure and internal flow fields of the flapper–nozzle mechanisms in hydraulic systems, research into improving their linearity through structural optimization when dealing with gas transmission media is lacking.

As pneumatic control systems develop, the air supply pressure continues to rise, and the compressibility of the gas inside nozzles during high-speed flow must be considered. However, flow field analysis for pneumatic servo control systems rarely considers this factor. Additionally, gas compressibility can introduce issues such as heat exchange, noise, vibration, and more [17,18]. These issues significantly affect the accuracy of pneumatic flapper–nozzle system simulations and limit their practical applications.

To address the issue of the short linear operating range of flapper–nozzle mechanisms, this paper attempts to introduce a negative-pressure flapper–nozzle structure. It utilizes the negative pressure generated inside the nozzle’s backpressure chamber to improve the linearity of the mechanism’s displacement-pressure relationship and enhance the pressure from the air source. Additionally, a convergent–divergent-type nozzle is applied inside the nozzle, and the internal flow field distribution and the relationship between pressure and flapper clearance are analyzed using simulations. Simulation selection employed the orthogonal experimental design method. Orthogonal experimental design is a research methodology for studying multiple factors and levels. It allows for the selection of representative combinations of levels from a comprehensive experiment for testing and analysis, ultimately identifying the optimal combination of levels [19]. The internal geometric parameters are optimized, a prototype is manufactured, and experimental validation is conducted.

The remainder of this paper is organized as follows: Section 2 introduces the working principle and mathematical modeling of the negative-pressure flapper–nozzle mechanism, explaining the design concept of the internal negative-pressure device and providing a preliminary selection of the mechanism through mathematical modeling. Section 3 discusses the preparation process for simulation and experiments, including the control equations and turbulence models used in the grid numerical simulation. To ensure that the results are independent of the mesh partition, grid independence verification is conducted for both the convergent–divergent-type throttle nozzle and the entire mechanism. The setup and preparation of the experimental platform are also described. Section 4 discusses the results of the simulation and experiments. By combining orthogonal experimental methods with numerical simulation, the selection of the throttle nozzle and the internal flow state analysis are determined and the simulation results are compared with the experimental results and discussed. Section 5 provides a summary of the research work in this paper.

## 2. System Design and Modeling

### 2.1. Flapper–Nozzle Mechanism Operating Principle

**Figure 1:**
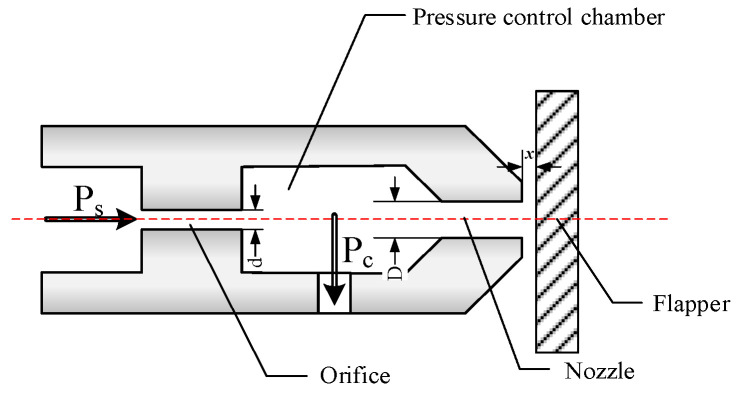
Flapper–nozzle mechanism. The flapper–nozzle mechanism consists of an orifice, a pressure control chamber, a nozzle, and a flapper connected in series. The pressure of the input compressed air from the air source is denoted as ‘*P_s_*’, and the pressure within the pressure control chamber is denoted as ‘*P_c_*’. The output air pressure is the same as the pressure within the pressure control chamber. The distance between the nozzle and the flapper is represented as ‘*x*’, the diameter of the orifice is represented as ‘*d*’, and the nozzle diameter is represented as ‘*D*’.

As illustrated in Figure 1, the flapper–nozzle mechanism is one of the most prevalent displacement-flow or displacement-pressure converters in pneumatic measurement and control [9]. Its role is to convert the minute displacement of the flapper into pressure changes within the pressure control chamber. The operational principle of the mechanism is as follows: compressed air from the air source enters the pressure control chamber through the main orifice on the left and flows from the gap between the nozzle and flapper to the atmosphere. The airflow path formed by the nozzle and flapper can be viewed as a variable throttle, and alterations in the gap (denoted as ‘*x*’) result in variations in the airflow passing through it. Under operational conditions, the gas pressure (air source pressure ‘*P_s_*’) entering the main orifice remains constant. Within a certain range of gap settings, when the flapper moves closer to the nozzle, the gas flow through the gap decreases, leading to an increase in pressure (*P_c_*) within the pressure control chamber. Conversely, when the flapper moves away from the nozzle, the pressure (*P_c_*) within the chamber decreases. Consequently, there is a one-to-one correspondence between the backpressure (*P_c_*) and the gap between the nozzle and the flapper. Therefore, precise control of the gap between the nozzle and the flapper allows for the modulation of pressure changes in the form of air pressure.

### 2.2. Working Principle of the Negative-Pressure Flapper–Nozzle Mechanism

**Figure 2:**
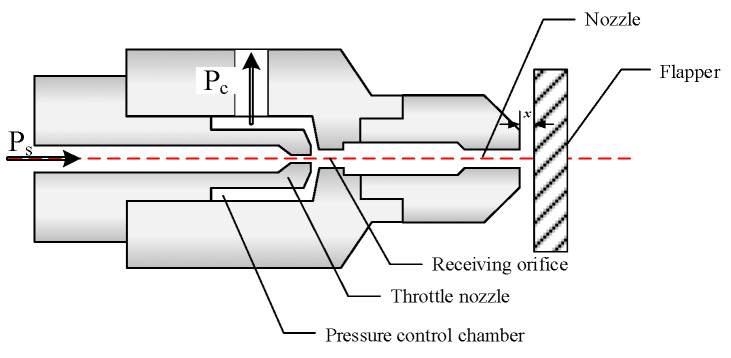
Negative-pressure flapper–nozzle system. The negative-pressure flapper–nozzle mechanism replaces the orifice with a nozzle structure of the same diameter. The pressure control chamber is connected to the throttle nozzle, allowing the system to generate a gas jet within the pressure control chamber during operation. The jet flows into the orifice and eventually exits from the gap between the nozzle and the flapper.

The negative-pressure flapper–nozzle structure, as shown in Figure 2, modifies the constant orifice into a nozzle structure while simultaneously altering the structure of the pressure control chamber. The orifice is now referred to as a throttle nozzle, and the jet flows into the receiving orifice in front after being discharged from the throttle nozzle.

**Figure 3:**
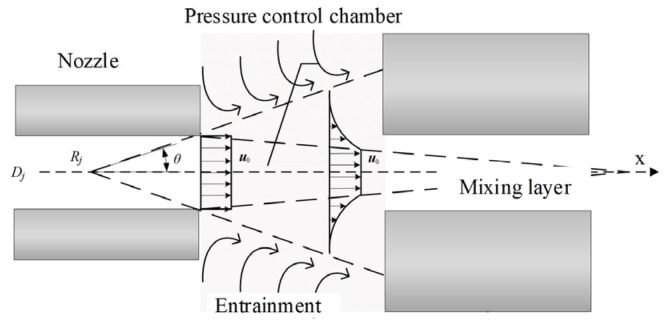
Velocity distribution in the pressure control chamber. The velocity distribution characteristics of the jet within the mixing region are as follows: the central portion constitutes the velocity core zone, where velocities are uniform; outside the velocity core zone, the flow velocity gradually decreases until it reaches zero at the boundary.

As shown in Figure 3, ignoring the impact of other structures on the jet’s state, the fluid ejected from the nozzle forms a velocity-discontinuity interface with the surrounding motionless gas due to its initial velocity. The fluid velocities on different interfaces vary and are discontinuous. According to turbulent fluid dynamics, the fluid on the velocity-discontinuity interfaces fluctuates and inevitably develops into vortices, leading to turbulence. Turbulence will entrain initially stationary gas into the jet, causing jet entrainment phenomena [20,21].

After the jet is discharged from the orifice, there exists a discontinuity interface between the main jet and the surrounding stagnant environmental fluid. Under the influence of viscous forces, the main jet continuously entrains the stagnant environmental fluid, leading to an increase in mass flow rate along the flow cross-section. By utilizing the entrainment effect generated by the orifice, and with appropriate structural adjustments, negative pressure (lower than 1 atm) can be generated within the pressure control chamber, forming the basis for the design of a negative-pressure nozzle.

The operational state of this mechanism is consistent with that of the flapper–nozzle mechanism. As the gap ‘*x*’ between the flapper and the nozzle increases, the backpressure in the pressure control chamber decreases. When the flapper approaches the nozzle, the backpressure in the pressure control chamber increases.

Because of the replacement of the orifice with a nozzle structure, a jet can be generated within the pressure control chamber. When the airflow exits the nozzle and enters the pressure control chamber, small air masses along the jet’s boundary start moving, causing the gas within the control chamber to be entrained into the jet. When the gap ‘*x*’ is sufficiently large, the internal jet results in the formation of negative pressure within the pressure chamber. When the gap between the nozzle and the flapper is within the operating range, the jet velocity generated by the throttle nozzle is relatively low, and its characteristic curve is similar to that of the traditional flapper–nozzle structure. As the flapper continues to move, the jet velocity increases, ultimately resulting in a chamber pressure lower than atmospheric pressure.

**Figure 4:**
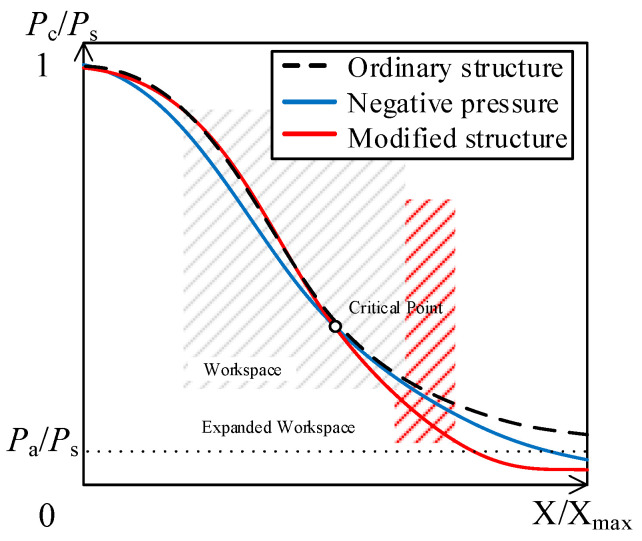
Comparison of characteristic curves. The curves in the figure represent the displacement-pressure characteristic curves of the mechanism. The black curve represents the flapper–nozzle mechanism, the blue curve represents the negative-pressure flapper–nozzle mechanism, and the red curve represents the modified negative-pressure flapper–nozzle mechanism with modifications to the throttle nozzle. The critical point indicates the state where the airflow reaches full expansion exactly at the exit of the convergent–divergent nozzle and *P*_a_ represents atmospheric pressure.

As shown in Figure 4, a system with acceptable performance should exhibit a nearly linear characteristic curve within the operating range, rather than displaying an “S” shape. In the case of traditional flapper–nozzle systems, the characteristic curve exhibits significant bending, and the only way to mitigate the impact of this curve is by reducing the workspace. However, the negative-pressure flapper–nozzle mechanism reduces the pressure output of the system after the critical point, resulting in a reduced degree of curvature in the characteristic curve. After further improvement of this structure, the system’s characteristic curve extends into the negative-pressure region, while still maintaining a higher-pressure output for smaller flapper displacements. This indicates that the new system possesses a wider operating range.

While changing the throttle orifice to a nozzle structure can increase the length of the linear range, the overall linearity improvement of the system may be limited. To enhance the system’s linearity, adjustments to the structure of the throttle nozzle are required. By examining the characteristic curve in Figure 4, it becomes evident that even for the negative-pressure nozzle, maintaining linearity becomes challenging when the gap ‘*x*’ is large. To mitigate its impact, it is advisable to keep the pressure drop minimal at small gap ‘*x*’ values, while in the latter part, the throttle nozzle can generate a strong jet, causing a rapid decrease in pressure within the control chamber. This shift in the nonlinear range to beyond the operating range helps improve system linearity.

### 2.3. Convergent–Divergent Throttle Nozzle

**Figure 5:**
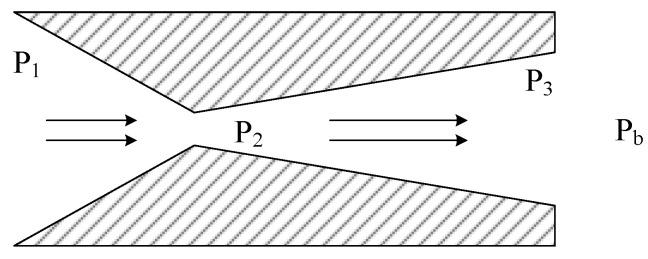
Convergent–divergent nozzle structure. The pressure when the airflow enters the convergent–divergent nozzle is *P*_1_, the pressure at the throat is *P*_2_, the pressure at the nozzle exit is *P*_3_, and the backpressure in the pressure control chamber is *P*_b_.

When high-pressure gas flows through the internal throttle nozzle, factors such as changes in the cross-sectional area and heat exchange with the pipe wall affect the flow. However, not all factors have the same impact on the internal flow. For the flow within the nozzle, changes in the cross-sectional area have the most significant influence, while the friction between the gas and the pipe wall is relatively minimal for the overall flow. For subsonic flows, the velocity change is inversely correlated with the cross-sectional area, while for supersonic flows, the gas accelerates in the converging section and reaches sonic velocity at the throat before further acceleration in the diverging section. In the operation of the flapper–nozzle mechanism, the flow state changes with the displacement between the flapper and the nozzle. By utilizing the relationship between velocity and cross-sectional area in subsonic and supersonic flows, a well-designed nozzle shape can result in a pressure gradient with low sensitivity in the initial stages of flapper movement, followed by an increased pressure gradient in the later stages. As shown in Figure 5, designing the nozzle as a convergent–divergent structure can accelerate the subsonic gas flow to supersonic in the contraction section, increase velocity at the throat to sonic velocity, and then continue to accelerate in the expansion section to achieve supersonic flow. This results in an increased negative pressure in the pressure chamber. The area ratio of the nozzle is determined by the diameter of the throat, as indicated in Section 2.4, and the nozzle structure needs to be designed based on the pressure ratio between the nozzle’s entrance and exit. 

### 2.4. Mechanism Modeling and Selection

**Figure 6:**
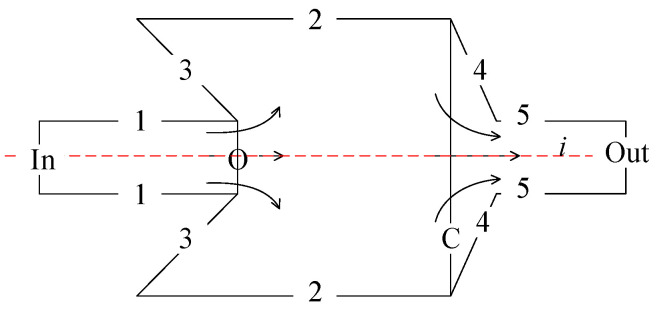
Upstream flow through the orifice and into the chamber of the flapper–nozzle system. Among them, 1, 2, 3, 4, 5, *O*, *In*, *Out* are the marks for each cross-section.

When the negative-pressure flapper–nozzle mechanism operates, the gap between the flapper and the nozzle is very small, and the internal flow speed of the nozzle is not sufficient to generate negative pressure in the pressure control chamber. By neglecting the impact of replacing the throttle orifice with a nozzle, which increases the gas flow velocity inside the control chamber, we can correlate the design of the negative-pressure nozzle to the traditional flapper–nozzle mechanism [22]. It is necessary to consider the compressibility of the fluid when the flow velocity exceeds 0.3 Mach. Due to the high gas pressure and the short length of the pipes, the flow is compressible adiabatic flow. The simplified mechanism is shown in Figure 6. First, it is assumed that the velocity is uniformly distributed in cross-sections *O* and *C*. In addition, it is assumed that the flow is steady and composed of an ideal gas. Under these conditions, *D* represents the volume between cross-sections *O*, 1, 2, 3, and *C*; *ρ* represents the density of the fluid; while *V* denotes the speed of the flow.

The continuity equation can be written as:

(1) $$\underset{D}{∭}div\left(\rho V\right)dD=0$$

Neglecting the volume forces acting on the ideal gas, the momentum conservation equation can be written as:

(2) $$\underset{D}{∭}div(\rho V⊗V+PI-\Sigma_{v})D=0$$

where *I* represents the unit tensor, *P* represents the pressure, and all pressures mentioned in this section are absolute pressures. $\Sigma_{v}$ represents the viscous stress tensor. Neglecting the impact of radiative heat, the total enthalpy equation can be represented as:

(3) $$\underset{D}{∭}div(\rho Vh_{i}-\Sigma_{v}\cdot V-\lambda \;gard\;T)dD=0$$

where *T* represents the temperature distribution in the system and *h_i_* represents stagnation enthalpy. The Reynolds number (Re) is used to determine whether the flow is laminar or turbulent. Under conditions of stable pressure, no external disturbances, smooth internal walls, and Re is less than 2300, the flow is laminar [23]. As Re increases, the flow gradually transitions to a turbulent state. When flow is turbulent within section *O*, the velocity profile can be represented as:

(4) $$V=V_{o}{\left(\frac{y}{r}\right)}^{\frac{1}{n}}$$

Turbulent flow typically occurs at a Reynolds number (Re) of around 2300, for simplification, we use the average flow velocity $\bar{V_{o}}$ to replace *V_o_*,

(5) $$\bar{V_{o}}=0.82V_{o}$$

For turbulent flow, the velocity distribution smoothens as Re increases, with *n* being approximately 7. The application of the divergence theorem can replace the original equation with a balance of fluxes on the surface. Considering that the control volume is relatively short in the *i*-direction, the flow in section *C* is within the mixing zone of the jet that flows out of section *O*, and assuming that section *C*’s length is greater than the thickness of the jet mixing zone, it can be written in the form of a normal distribution. The velocity distribution in section *C* can be defined as:

(6) $$\frac{V_{c}}{V_{o}}=exp\left[-{\left(\frac{y-b_{e}}{b_{m}}\right)}^{2}\right]$$

where *b_e_* is the semi-thickness of the core region and *b_m_* is the thickness of the mixing zone. The average velocity in section *C* can be written as:

(7) $$\bar{V_{c}}=b_{e}\sqrt{\pi b_{m}^{2}}$$

On walls 1, 2, and 3, where no fluid enters or exits, the continuity equation can be written as:

(8) $$-\iint \rho_{o}\bar{V_{o}}S_{o}dx_{o}dy_{o}+\iint \rho_{c}\bar{V_{c}}S_{c}dx_{c}dy_{c}=0$$

where *x* represents the length of the inlet section *O* along the *i*-axis, and *y* denotes the distance measured from the centreline in the vertical direction. We introduce Mach numbers *M_o_* and *M_c_*, stagnation pressures *Pi_o_* and *Pi_c_*, and temperatures *Ti_o_* and *Ti_c_* in sections *O* and *C*. Where *Pi_j_* represents stagnation pressure in section *j*, *Pi_j_* represents stagnation temperature in section *j* According to the ideal gas equation and the speed of sound equation, leading to:

(9) P=ρRT

(10) $$a^{2}=\gamma RT$$

where *R* is the gas constant. For ideal gases, the ratio of *P*/*Pi* and *T*/*Ti* can be expressed in terms of Mach number and ratio of specific heats *γ* as follows:

(11) $$\frac{P}{Pi}=w\left(M\right)={\left(1+\frac{\gamma -1}{2}M^{2}\right)}^{-\gamma /\left(\gamma -1\right)}$$

(12) $$\frac{T}{Ti}=\theta \left(M\right)={\left(1+\frac{\gamma -1}{2}M^{2}\right)}^{-1}$$

The continuity equation can be expressed as follows:

(13) $$-\frac{w\left(M_{o}\right)}{\sqrt{\theta \left(M_{o}\right)}}M_{o}\frac{Pi_{o}\;}{\sqrt{Ti_{o}}}S_{o}+\frac{w\left(M_{c}\right)}{\sqrt{\theta \left(M_{c}\right)}}M_{c}\frac{Pi_{c}\;}{\sqrt{Ti_{c}}}S_{c}=0$$

(14) $$-\left(\rho_{o}{\bar{V_{o}}}^{2}+P_{o}\right)S_{o}-\iint_{1\cup 2}(\Sigma_{v}\cdot n)\cdot idS+\iint_{3}Pn\cdot ids+\left(\rho_{c}{\bar{V}}_{c}^{2}+P_{c}\right)S_{c}=0$$

where *S_j_* represents the area of section *j* and *n* represents the unit normal vector to the flow field *D*, pointing outward from the flow field. Since there are no vorticities on surfaces 1, 2, and 3, and assuming that the flow in sections *O* and *C* is uniform, the entire flow field is symmetric about the *i*-axis, and asymmetrical flow in the flow field is ignored. The momentum conservation equation along the *i*-axis is as follows:

The integral terms for surfaces 1 and 2 represent the frictional forces generated by these surfaces. Neglecting the wall frictional forces and introducing the Mach numbers, it can be written as:

(15) $$-\sigma \left(M_{o}\right)w\left(M_{o}\right)Pi_{o}S_{o}-P_{b}\left(S_{c}-S_{o}\right)+\sigma \left(M_{c}\right)w\left(M_{c}\right)Pi_{c}S_{c}=0$$

The function defined in terms of the Mach number is:

(16) $$\sigma \left(M\right)=1+\gamma M^{2}$$

For compressible adiabatic flow, the total enthalpy equation can be simplified to:

(17) $$Ti_{o}=Ti_{c}$$

For viscous fluids, the sudden expansion or contraction of a cross-section leads to a local decrease or increase in pressure, and this phenomenon is exacerbated by the presence of friction. When the flow’s Reynolds number (Re) is sufficiently high, the pressure drop due to friction is much lower compared to the local pressure drop. For the sake of convenience in calculations, the fluid is treated as an ideal gas and the fluid exiting the jet nozzle is considered to be uniform, leading to:

(18) $$-\frac{w\left(M_{o}\right)}{\sqrt{\theta \left(M_{o}\right)}}M_{o}Pi_{o}S_{o}+\frac{w\left(M_{c}\right)}{\sqrt{\theta \left(M_{c}\right)}}M_{c}Pi_{c}S_{c}=0$$

(19) $$-\sigma \left(M_{o}\right)w\left(M_{o}\right)Pi_{o}S_{o}-w\left(M_{o}\right)Pi_{o}(S_{c}-S_{o})+\sigma \left(M_{c}\right)w\left(M_{c}\right)Pi_{c}S_{c}=0$$

For sections *C* and *F*, the continuity equation can be written as:

(20) $$\frac{w\left(M_{c}\right)}{\sqrt{\theta \left(M_{c}\right)}}M_{c}\frac{S_{c}}{S_{f}}=\frac{w\left(M_{f}\right)}{\sqrt{\theta \left(M_{f}\right)}}M_{f}$$

Substituting $P_{a}=Pi_{c}w\left(M_{f}\right)$ into Equation (18), we obtain:

(21) $$\frac{\sqrt{\theta \left(M_{f}\right)}}{M_{f}}=\frac{\sqrt{\theta \left(M_{o}\right)}}{w\left(M_{o}\right)M_{o}}\frac{S_{f}}{S_{o}}\frac{P_{a}}{Pi_{o}}$$

Combining Equations (1) and (2), we can obtain the equation describing the pressure drop as follows:

(22) $$\frac{\sigma \left(M_{c}\right)\sqrt{\theta \left(M_{c}\right)}}{M_{c}}=\frac{\sigma \left(M_{o}\right)\sqrt{\theta \left(M_{o}\right)}}{M_{o}}\left[1+\frac{\frac{S_{c}}{S_{o}}-1}{\sigma \left(M_{o}\right)}\right]$$

When solving the system for Equations (20)–(22), there are three unknowns: the gas flow Mach numbers *M_o_*, *M_c_*, and *M_f_*. In the case of subsonic flow (*M_o_* = *M_f_* = 1 and *M_c_* < 1), the pressure distribution inside the control chamber *P*_c_ can be expressed as:

(23) $$P_{c}=Pi_{o}\omega \left(M_{o}\right)\frac{\sqrt{\theta \left(M_{c}\right)}}{\sqrt{\theta \left(M_{o}\right)}}\frac{M_{o}S_{o}}{M_{c}S_{c}}$$

**Figure 7:**
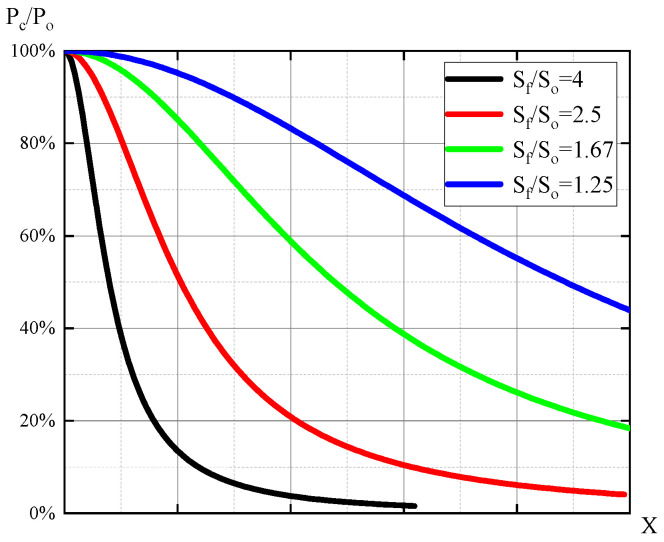
Flapper–nozzle characteristic curves. Sf represents the flow area of the nozzle, and so represents the flow area of the throttle. The black curve represents the system’s characteristic curve for *S_f_*/*S_o_* = 1.25, the red curve represents *S_f_*/*S_o_* = 2.5, the green curve represents *S_f_*/*S_o_* = 1.67, and the blue curve represents *S_f_*/*S_o_* = 4.

According to Equation (23), the pressure change curve of *P*_c_ as a function of flapper displacement 0-*X*_max_ is shown in Figure 7. It can be observed that with the increase in flapper displacement *x*, the pressure in the control chamber *P*_c_ gradually decreases. The sensitivity of the flapper–nozzle system increases with the increase in *S_f_*/*S_o_*. On the right side of the solid line in the graph, the curve is relatively flat, indicating that when *D* is a constant value, further increasing *x* has little impact on backpressure. From the characteristic curve, it is evident that as the ratio (*S_f_*/*S_o_*) increases, the influence of *x* displacement on backpressure becomes more significant. This leads to reduced control stability and a higher likelihood of oscillations, but it offers high sensitivity and lower air consumption, with lower demands on driving power. Conversely, when the ratio is smaller, the influence of *x* displacement on backpressure decreases, resulting in better control stability but slower control speed and higher energy consumption. This requires higher driving power. Based on the flapper–nozzle characteristic curve and engineering requirements, the diameter of the orifice is determined to be 0.3 mm, and the nozzle diameter is determined to be 0.5 mm.

## 3. Simulation Methods and Experimental Preparation

### 3.1. Governing Equations

The flow within the flapper–nozzle mechanism exhibits turbulent behavior. While a direct numerical solution to the Navier–Stokes (N–S) equations can provide detailed insights into turbulent flow, it is computationally demanding in terms of memory and time requirements. When the objective is to predict the average scalar field, velocity field, and turbulence-induced forces, the N–S equations can be subjected to an averaging process. This leads to the formulation of the Reynolds-Averaged Navier–Stokes (RANS) equations, which can be represented as follows [23]:

(24) $$\frac{\partial \rho }{\partial t}+\frac{\partial }{\partial x_{i}}\left(\rho u_{i}\right)=0$$

(25) $$\frac{\partial }{\partial t}(\rho u_{i})+\frac{\partial }{\partial x_{j}}\left(\rho u_{j}u_{i}\right)=-\frac{\partial p}{\partial x_{i}}+\frac{\partial \sigma_{ij}}{\partial x_{j}}+\frac{\partial }{\partial x_{j}}\left(-\rho u_{i}u_{j}^{\prime }\right)$$

where *u_i_* represents the Reynolds mean velocity component with the mean symbol omitted, *ρ* is the density, *p* is the pressure, *u* is the pulsation velocity, and σ is the stress tensor component.

The RANS equations introduce additional terms associated with Reynolds stress, which account for the effects of turbulence. However, these additional terms render the RANS equations non-closed. To maintain the same number of equations as unknowns and satisfy the theoretical solution conditions, a turbulence model must be incorporated. Turbulence models estimate the impact of small-scale turbulence on large-scale flow without explicitly solving the N–S equations. 

### 3.2. Turbulence Models (Two-Equation Model)

The *k-ε* turbulence model relies on empirical coefficients determined through simple shear flow experiments. However, when treating Reynolds stress, it assumes isotropic turbulence, which can result in significant discrepancies between numerical simulations and experimental results, especially in regions with high strain rates. The internal jet generated by the flapper–nozzle system exhibits specific characteristics such as curved streamlines and recirculation near the backpressure chamber and the receiving orifice. The standard *k-ε* turbulence model may lead to substantial errors in predicting the flow field. To address these limitations, the RNG *k-ε* turbulence model, which shares a similar structure with the standard *k-ε* model, is employed. It utilizes the Boussinesq eddy viscosity hypothesis to close the Reynolds stress term. Additionally, it incorporates terms associated with rotation in the dissipation rate equation and applies the Reynolds analogy for turbulent diffusion in scalar fields. This model is particularly well-suited for handling flow near the wall in impinging jet flows. The transport equations for turbulent kinetic energy (*k*) and dissipation rate (*ε*) in the RNG *k-ε* turbulence model can be expressed as follows [24]:

(26) $$\frac{\partial \left(\rho k\right)}{\partial t}+\frac{\partial \left(\rho ku_{i}\right)}{\partial x_{i}}=\frac{\partial }{\partial x_{i}}\;\left[\;a_{k}\mu_{eff}\frac{\partial k}{\partial x_{i}}\right]+G_{k}-p\varepsilon$$

(27) $$\frac{\partial \left(\rho z\right)}{\partial t}+\frac{\partial \left(\rho \varepsilon u_{i}\right)}{\partial x_{i}}=\frac{\partial }{\partial x_{i}}\left[a_{\varepsilon },\mu_{eff}\frac{\partial \varepsilon }{\partial x_{i}}\right]+C_{1\varepsilon }^{*}\frac{\varepsilon }{k}G_{k}-C_{2\varepsilon }\rho \frac{\varepsilon^{2}}{k}$$

In these equations, $\mu_{eff}=\mu +\mu_{t}$ represents the effective viscosity coefficient; $\mu_{t}=\rho C_{\mu }\mu k^{2}/\varepsilon$ is the turbulence viscosity coefficient; *μ* stands for dynamic viscosity; *C_μ_* is the turbulence model constant; $G_{k}=\mu_{t}(\partial u_{i}/x_{j}+\partial u_{j}/x_{j})*\partial u_{i}/x_{j}$ corresponds to turbulent energy due to the mean velocity gradient; $\alpha_{c}=\alpha_{k}=1.39$ are the reciprocals of the effective Prandtl numbers for ε (turbulence dissipation rate) and k (turbulence kinetic energy), respectively; $C_{1\varepsilon }^{*}=C_{1\varepsilon }-\eta (1-\eta )/(1+\eta^{3})$, $C_{1k}^{*}=C_{1k}-\eta (1-\eta )/(1+\beta \eta^{3})$, $\eta =\sqrt{2S_{ij}S_{ij}}k/\varepsilon$; $S_{ij}=(\partial u_{i}/x_{j}+\partial u_{j}/x_{i})/2$, represent the Reynolds stress components; the other constants used in the RNG *k-ε* model equations are given by $C_{\mu }=0.0845$, $C_{1\varepsilon }=1.42$, $C_{1k}=1.68$, η=4.38, β=0.012.

### 3.3. Grid Generation and Grid Independence Verification for Orifice Flow

In Section 2.2, it was discussed that the flow state inside the nozzle orifice undergoes continuous changes as the gap between the flapper and the nozzle increases. To determine the optimal shape of the throttle nozzle passage, Computational Fluid Dynamics (CFD) simulations are employed for the contraction–expansion-type throttle nozzle. The flow state inside the orifice is primarily influenced by the inlet pressure (*P*_1_) and the outlet pressure (*P*_3_). Considering the symmetry of the fluid computational domain and focusing on local flow conditions, a 2D model of the throttle orifice is developed by simulating the effect of flapper motion on the gas flow state through variations of the outlet pressure. ANSYS ICEM 2022 R1 is used to partition the computational fluid domain into a rectangular grid, with grid sizes adjusted based on the flow characteristics inside the orifice. Local refinements are applied to the flow boundaries, the throat of the convergent–divergent passage, and the orifice outlet. In regions where flow variations are less significant, the grid is set to be sparser and more uniform.

**Figure 8:**
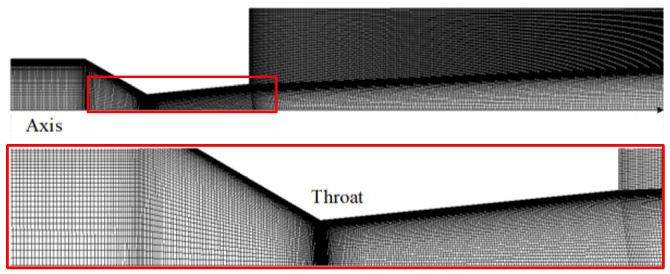
Grid division result of the convergent–divergent-type throttle nozzle. To facilitate the observation of the grid structure, the area within the upper red-brown box has been proportionally enlarged. The magnified image is located within the lower red box.

To ensure accuracy and eliminate the influence of the grid on the numerical calculation results, grid independence validation is conducted using six different grid schemes for a nozzle with a throat diameter of 0.3 mm, a contraction angle of 30°, and an expansion angle of 5°. The grid details are presented in Table 1 and Figure 8.

**Figure 9:**
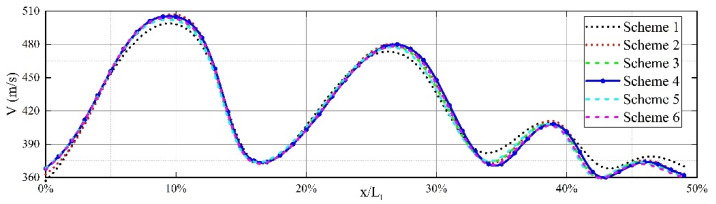
Jet axial velocity distribution in front of the nozzle. The six curves represent six different grid partitioning schemes, with Scheme 4 applied in subsequent calculations.

Six different grid models were simulated individually, and for high Reynolds number flows, the RNG k-ε turbulence model was found to yield higher computational accuracy. The evaluation criterion for this study, focusing on the flow, is the velocity. The influence of the six grid schemes on the computed results at the same location in the flow field is analyzed. The maximum values of velocity and turbulent kinetic energy along the jet axis are extracted from the nozzle exit. From Figure 9, it can be observed that, except for scheme 1 and scheme 2, the velocity distribution along the jet axis under different grid schemes exhibits good consistency, indicating that grid density has a relatively small impact on the flow field distribution. Therefore, to save computational resources while ensuring computational accuracy, Scheme 4 is selected for grid partitioning in all computational models.

### 3.4. Grid Generation and Grid Independence Verification for Internal Nozzle Flow

The entire negative-pressure flapper–nozzle assembly was modeled for simulation purposes, with the aim of investigating the relationship between the internal nozzle jet velocity and the pressure within the backpressure chamber. This information would guide the selection of the optimal internal nozzle structure to achieve the maximum negative pressure within the backpressure chamber. Additionally, the effect of the distance between the internal nozzle and the receiving orifice on the backpressure was explored. A final model was constructed and simulated for the selected flapper–nozzle assembly to determine the relationship curve between the flapper gap and the backpressure. To ensure the universality of grid independence verification, a 3D model of the complete negative-pressure flapper–nozzle assembly was selected for grid generation and independence verification. The grid generation processes for other simulation setups have been omitted for brevity.

To simulate the movement of the flapper, dynamic mesh techniques were employed to handle the moving boundary problem. In the context of the flapper–nozzle assembly, the boundary motion was predefined and implemented using User-Defined Functions (UDFs). Due to the irregular shapes of various parts of the fluid domain and the inclusion of both dynamic and static regions in the calculation domain, areas experiencing deformation during internal flow simulations required the application of dynamic mesh for simulation. The dynamic mesh necessitated the use of tetrahedral grids, and ANSYS MESHING was utilized to generate the tetrahedral grid for the fluid domain. In the region between the nozzle jet outlet and the receiving orifice, where primary suction occurred, a finer grid was implemented to ensure high-precision modeling of the suction process. Furthermore, the grid expansion rate was reduced to concentrate the majority of the grid elements in the core area of the internal jet and the impact area of the receiving orifice. Considering the symmetry of the flow domain, only half of the flow domain was simulated.

**Figure 10:**
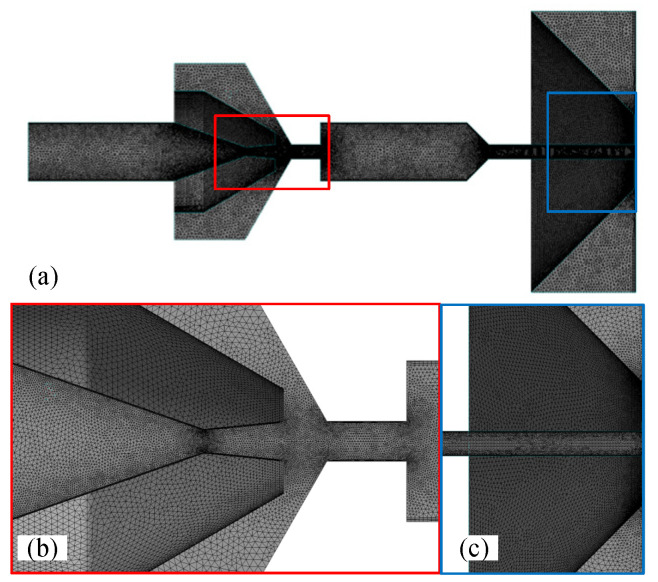
Computational domain grid of the flapper–nozzle system. (**a**) shows the grid division of half of the internal flow field of the assembly. To facilitate observation, the grid area around the nozzle orifice has been proportionally enlarged. (**b**,**c**) enlarge the details of the corresponding color area displayed.

To ensure accuracy and eliminate the influence of grid factors on numerical simulation results, mesh-independent verification was conducted using six different grid schemes. The model used for verification comprised a nozzle with a throat diameter of 0.3 mm, a contraction angle of 30°, an expansion angle of 5°, a receiving orifice with a diameter of 0.5 mm and a length-to-diameter ratio of 2, and a flapper–nozzle assembly with a 0.5 mm gap between the throttle nozzle and the receiving orifice, unobstructed by the flapper. All grid schemes employed tetrahedral meshing, and the number of mesh elements increased progressively. The grid division details are provided in Table 2 and Figure 10.

**Figure 11:**
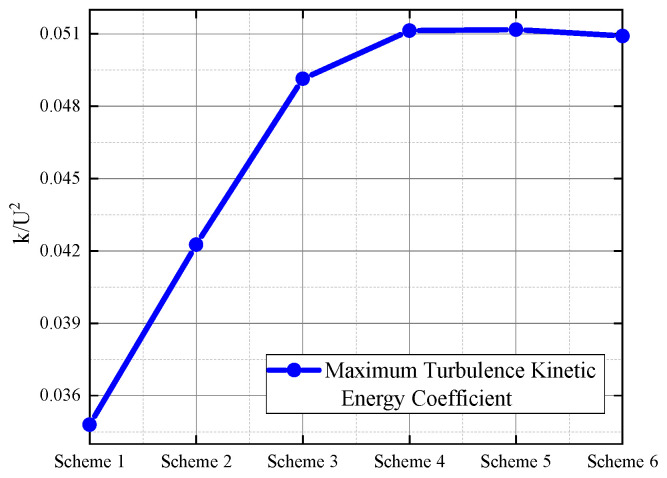
Turbulence kinetic energy distribution. The blue solid line represents the maximum turbulence kinetic energy coefficient within the computational domain obtained after each grid division, gradually converging to 0.051 as the grid is refined.

For high Reynolds number flow, the RNG *k-ε* turbulence model was applied to compute the geometrical models of the six grid schemes. The evaluation of interest in this study focused on flow velocity and turbulence kinetic energy. The impact of these 6 grid schemes on the calculation results at the same position in the flow field was analyzed. The maximum turbulence kinetic energy coefficient in the flow field was extracted at the exit of the orifice for comparison. As shown in Figure 11, the maximum turbulence kinetic energy coefficient in the flow field steadily increases with the increase in grid density. Starting from scheme 3, further grid refinement has almost no effect on the maximum turbulence kinetic energy coefficient. It can be observed that turbulence kinetic energy is highly sensitive to grid density, but it converges after reaching the grid density of scheme 4. Further, increasing grid density does not change the turbulence kinetic energy calculation results; thus, to save computational resources while ensuring accuracy, scheme 4 was chosen for grid division in all computational models.

### 3.5. Experimental Design

**Figure 12:**
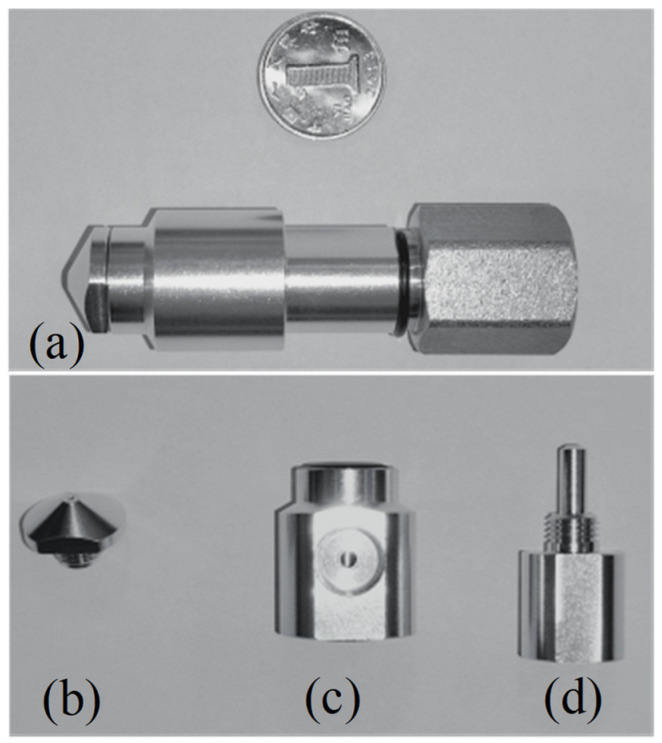
The negative-pressure nozzle, referred to as (**a**) the nozzle mechanism, is composed of three parts, namely (**b**) nozzle, (**c**) backpressure chamber, and (**d**) throttling nozzle, which maintain fine air tightness and facilitate processing.

**Figure 13:**
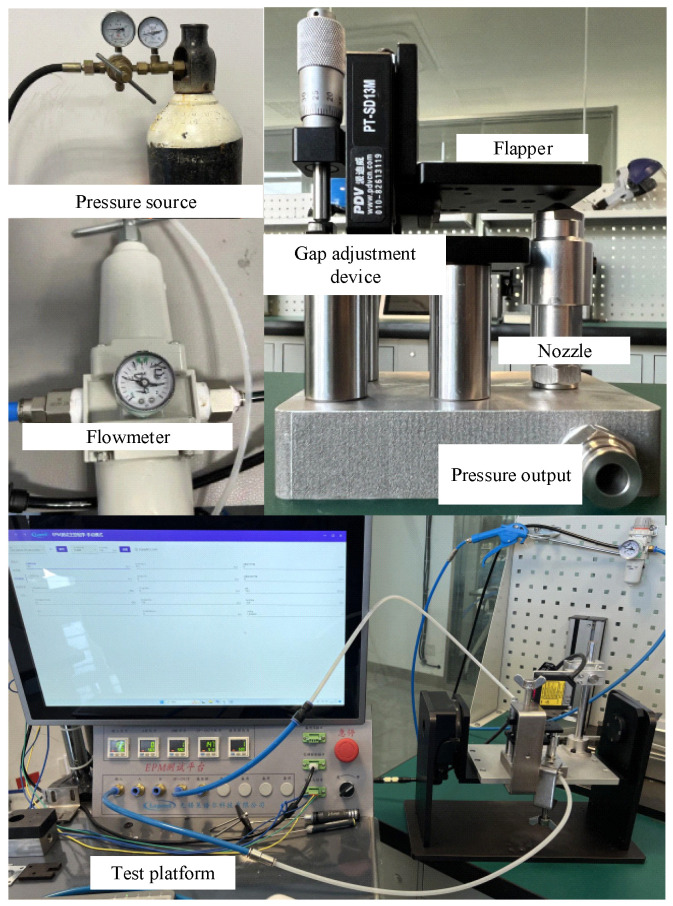
Experimental bench for generating the characteristic curve of the negative-pressure flapper–nozzle mechanism.

To validate the theoretical calculations and simulation results, an experimental setup, namely a flapper–nozzle displacement-pressure test bench, was constructed for this study. The prototype is depicted in Figure 12 and the experimental bench is illustrated in Figure 13. The primary components of the experimental bench include an air pressure source, flow meter, flapper–nozzle device, and data acquisition system. The flow meter integrates functions such as air pressure adjustment, measurement, and flow measurement. The initial gap between the nozzle and the flapper is determined by a spiral structure. The time interval of the experiment is measured and collected by a laser-ranging instrument, and the gap between the flappers is adjusted by a spiral device driven by a stepper motor. The test bench is employed to gather real experimental data, including the gap between the flappers, backpressure chamber pressure, and flow rate. The experimental setup comprises several components, including a high-pressure air source, a pressure regulator, a flow meter, a flapper–nozzle device, a pressure gauge, and a data acquisition system. The high-pressure air source, capable of a maximum output pressure of 15 MPa, supplies clean and dry high-pressure air to the flapper–nozzle. Following its release from the high-pressure air source, the gas enters the pressure regulator, which adjusts the gas pressure to 0.4 MPa. Subsequently, the gas enters the flow meter to record flow information before entering the flapper–nozzle mechanism. The data acquisition system is connected to the backpressure chamber of the flapper–nozzle mechanism to collect pressure data.

## 4. Results and Discussion

### 4.1. Numerical Analysis of the Orifice

In this study, the orifice plays an indispensable role, with its appropriate selection significantly enhancing system linearity. The orifice not only throttles the flow medium from the high-pressure gas source, generating sufficient pressure in the backpressure chamber, but it also utilizes its jet to regulate the backpressure chamber pressure by entraining the surrounding gas, thereby maintaining the entire system’s excellent linearity within the operational range. This section primarily focuses on the influence of geometric parameters on the jet state of the convergent–divergent nozzle, based on the characteristics of supersonic gas flow.

**Figure 14:**
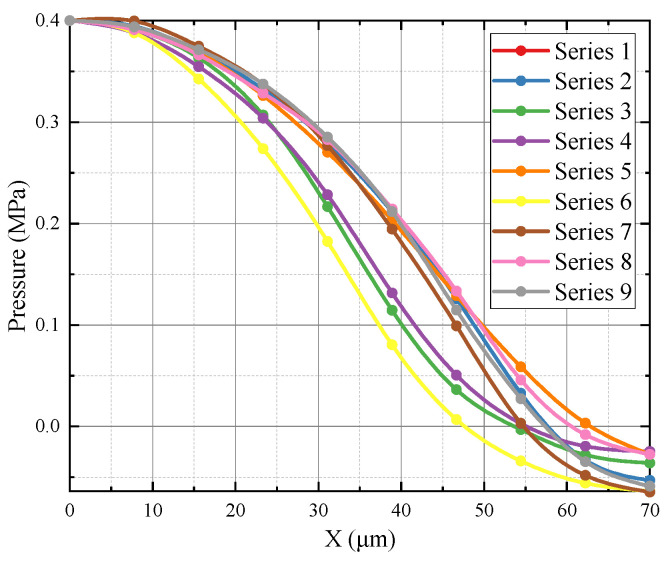
Orthogonal numerical simulation results. Each curve represents the characteristic curve of an orthogonal experimental setup, and each point represents a numerical simulation calculation. The characteristic curve is an important indicator for evaluating the performance of the setup.

The convergent section of the orifice has throat angles set at 15°, 30°, and 45°, with lengths of 0.5 mm, 1 mm, and 1.5 mm. The divergent section angles are set at 0°, 5°, and 10°, with lengths of 1.5 mm, 2.5 mm, and 3 mm. An orthogonal experimental design is employed. This is a method for studying multi-factor and multi-level designs, which selects representative points from a comprehensive experiment based on orthogonality. These representative points possess characteristics of “uniform dispersion and comparability”. Orthogonal experimental design is a primary method for analyzing factorial designs and an efficient, rapid, and economical experimental design method [25]. The orifice selection study is a 4-factor, 3-level experiment, which requires 4^3^ = 64 combinations of experiments without considering the repetition of each combination. As shown in Table 3, by utilizing the L9(34) orthogonal table for experiment arrangements, only nine experiments are needed, significantly reducing the workload. The flapper displacement is moved from 10 µm to 100 µm, with simulations conducted every 10 µm and the backpressure chamber’s internal pressure recorded. Simulations are conducted for test series 1–9, and the results are shown in Figure 14. Figure 14 illustrates the orthogonal numerical simulation results. Series 3, 4, and 6 are not effective at suppressing jet development at small clearances. Series 2, 7, and 9, although effective at inhibiting jet expansion at small clearances, prematurely reach a critical state inside the jet, leading to excessive pressure drop at the throat of the characteristic curve and making it non-linear. Series 1, 5, and 8 are closer to the ideal characteristic curve. Considering practical production difficulties and other factors, Series 5’s structure is selected as the orifice.

**Figure 15:**
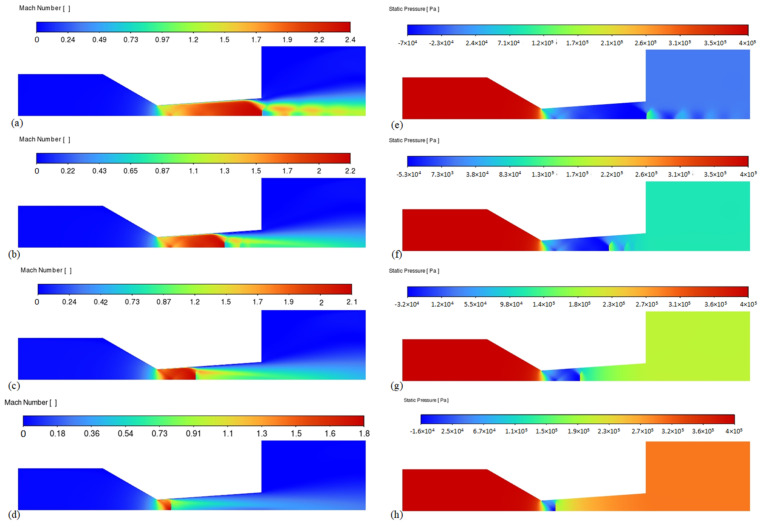
The effect of backpressure variation at the outlet on the internal flow state of the convergent–divergent nozzle. (**a**–**d**) show the jet velocity distribution and (**e**–**h**) present the jet pressure distribution. These simulations cover a range of backpressure conditions increasing from 0 to 0.3 MPa, from top to bottom.

The flow state of the convergent–divergent nozzle primarily depends on the geometric shape of the flow channel and the pressure difference between the inlet and outlet. To conserve computational resources and obtain accurate simulation results, it is necessary to simplify the flow field model. The flow region near the nozzle is extracted, with a pressure inlet at the inlet end matching the high-pressure gas source and a pressure-adjustable outlet at the outlet. Only the influence of flapper displacement on the backpressure at the nozzle is considered, and the movement of the flapper is simulated by adjusting the pressure at the outlet, thus simulating the flow state at the nozzle. To further investigate the internal flow state of the nozzle structure labeled as series 5, simulations were conducted under different backpressure conditions (0 MPa, 0.1 MPa, 0.2 MPa, 0.3 MPa). The simulation results, as shown in Figure 15, display the distribution of flow Mach numbers on the left and the pressure distribution on the right, corresponding to the velocity distribution on the left. The flow velocity variation observed in the simulation results of the convergent–divergent nozzle is generally consistent with theoretical analysis.

When the backpressure is 0 MPa, the airflow accelerates at the throat of the nozzle, forming two intersecting oblique shockwaves. The airflow continuously accelerates in the expansion section, reaching a maximum velocity of 2.4 Mach, but it does not achieve complete expansion at the outlet. After exiting the nozzle, a series of expansion waves are formed and the airflow continuously accelerates before the expansion waves, while the pressure continuously decreases. After passing through the expansion waves, it suddenly decelerates, causing a pressure increase; this process occurs repeatedly. With increasing backpressure, the transverse shockwave at the outlet continuously moves toward the interior of the nozzle until it reaches the throat. Correspondingly, the jet velocity generated by the nozzle gradually decreases. When the backpressure is 0.1 MPa, the gas at the nozzle outlet (just) reaches a fully expanded state, with the pressure nearly matching the backpressure. Supersonic flow is observed throughout the entire range of backpressure variation, even when the backpressure is 0.3 MPa, and expansion waves appear at the nozzle throat.

**Figure 16:**
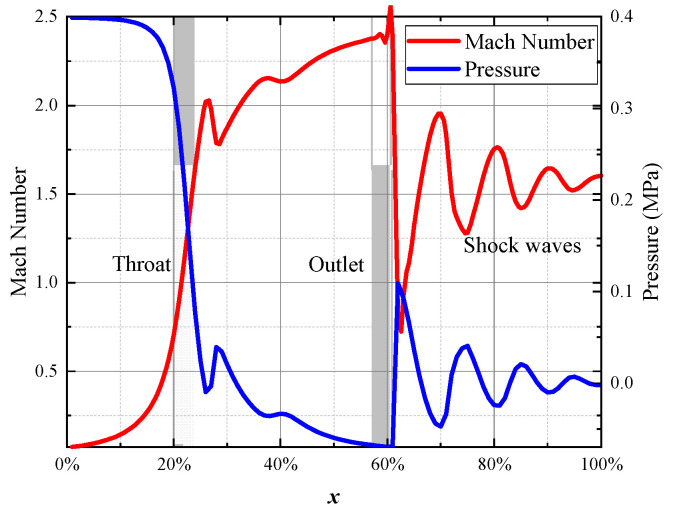
Velocity and pressure distribution during the shock wave. The red curves represent the simulation results for the internal flow velocity along the axis in the nozzle. The corresponding blue curves represent the simulation results for the pressure distribution along the same axis.

**Figure 17:**
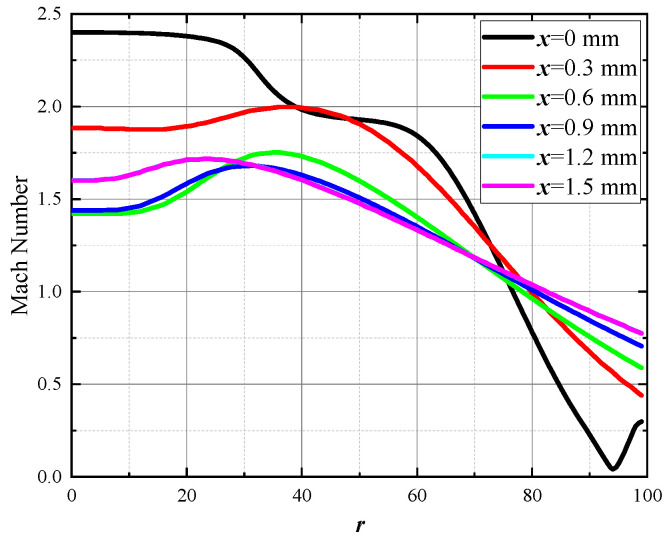
Radial velocity distribution. The curves in the figure represent the simulated radial velocity distribution of the jet within a range of 0–1.5 mm from the nozzle outlet. Simulations were conducted at intervals of 0.3 mm.

In Figure 16, when there is no flapper, and considering compressibility, the axial velocity distribution of the internal flow shows the following characteristics: Subsonic flow accelerates as it enters the contraction channel, causing a continuous drop in pressure until it reaches sonic conditions at the throat. Upon entering the expansion channel, subsonic flow accelerates to supersonic speeds and continues to accelerate, causing a drop in pressure. At the exit, the flow is not yet at a fully expanded state and generates a shock wave, leading to a sudden decrease in velocity and a rapid increase in pressure. Subsequently, a series of expansion waves form in front of the exit. Figure 17 shows the radial velocity distribution in the flow direction after the jet is discharged from the nozzle. As the jet develops, its volume gradually increases; however, the flow field does not exhibit a distribution similar to that of an incompressible jet. The flow velocity is relatively consistent in the region near the axis. There is some velocity fluctuation due to the presence of expansion waves; however, as the jet progresses, the overall trend is that of a decrease in velocity. After a certain distance, velocity begins to decrease with increasing distance from the axis, reaching subsonic speeds about 0.8 R away from the axis.

### 4.2. Negative-Pressure Flapper–Nozzle Characteristic Curve Simulations and Experimental Analysis

Based on the calculations and simulations described above, the final geometric parameters of the negative-pressure flapper–nozzle structure are as follows: the internal throttle orifice has a diameter of 0.3 mm, a contraction angle of 30°, a length of 1 mm, an expansion angle of 5°, and a length of 1.5 mm. The nozzle diameter is 0.5 mm, with an aspect ratio of 6. The receiving orifice has the same diameter as the nozzle and is located 0.5 mm away from the throttle orifice.

**Figure 18:**
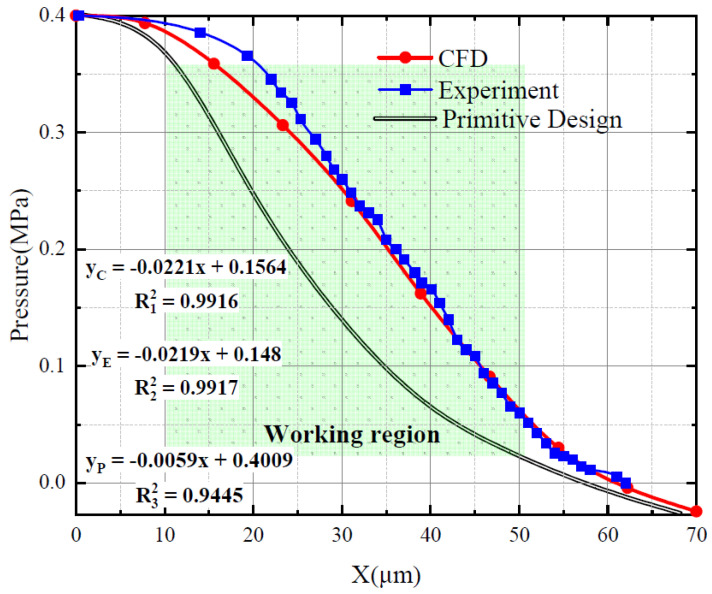
Negative-pressure flapper–nozzle mechanism characteristic curve simulation and experimental results. The red curve represents the numerical simulation results of the mechanism. Simulations were conducted for each 10-μm increase in the gap, and these results are indicated by the red curve. The blue curve represents the numerical simulation results of the mechanism, with simulations conducted for each 5-μm increase in gap.

Numerical simulations and experimental calculations were performed; the results are shown in Figure 18. The red curve represents the results obtained from the numerical simulations. Experiments were conducted on the primitive flapper–nozzle system and its characteristic curve was obtained (black line in the figure). The geometric modeling and grid partitioning are the same as in Section 3.4, and due to computational accuracy considerations, a simulation calculation was performed for every 10 μm of flapper movement. The blue curve represents the experimental values of pressure inside the backpressure chamber. The nozzle was fixed in front of the flapper, and the gap length between the nozzle and the flapper was adjusted using a spiral device. Experiments were conducted at 5-μm intervals to obtain the displacement-pressure characteristic curve for the system.

Both the numerical simulation and experimental results exhibit good linearity within the working range. A linear fit was performed for a portion of the working range, and the slopes of the resulting lines were roughly similar. For the fluid simulation results, the coefficient of determination (R-squared) for the fit is 0.9916; for the experimental results, it is 0.9917, and for the primitive flapper–nozzle, it is 0.9945. Both the simulation and experimental results maintained a high degree of consistency. This indicates that for nozzles and flapper valves used in high-pressure systems, simplifying the internal flow as adiabatic isentropic flow with the fluid treated as an ideal gas results in highly reliable simulation outcomes.

**Figure 19:**
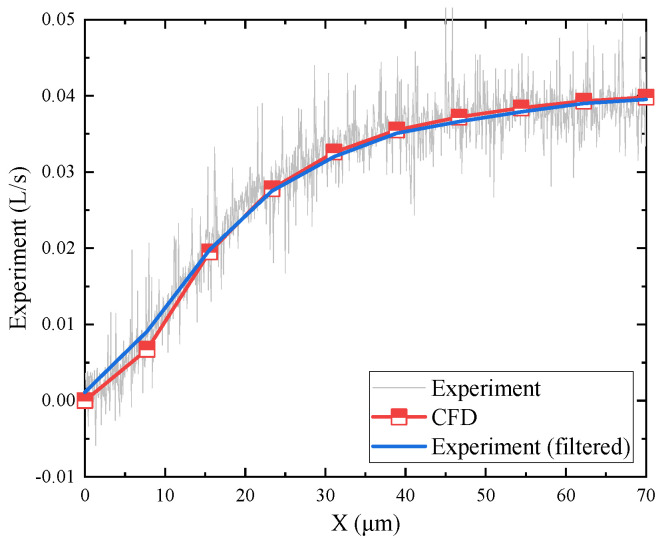
Negative-pressure flapper–nozzle mechanism flow simulation and experimental results. The red curve represents the numerical simulation results of the mechanism. Simulations were conducted for each 10-μm increase in the gap, and these results are indicated by the red curve. The gray curve represents the flow data collected by the flow meter. The blue curve is the result obtained after filtering and fitting the gray curve to eliminate noise.

The experimental and simulation results of flow rate changes during the operation of the mechanism are shown in Figure 19. The grey curve in the figure represents the flow rate data collected using a flow meter. The blue curve represents the data obtained after filtering and noise reduction. The red broken line represents the simulation results calculated every 10 μm of flapper displacement. Compared to the simulation results, the experimental data show minor gas flow, even when the flapper is not moving. Nevertheless, the overall trend of flow rate change is similar in both cases, with larger variations when the flapper displacement is small. The flow rate curve is quite steep at the initial stage, but as the flapper displacement continues to increase, the flow rate curve gradually becomes smoother.

## 5. Conclusions

In the present study, a novel flapper–nozzle mechanism intended for pneumatic valve positioners was developed, wherein the air source pressure was amplified to 0.4 MPa. A convergent–divergent nozzle was incorporated within the internal throttling hole, thereby facilitating the generation of supersonic jet flows within the nozzle. This innovative design utilizes the flow characteristics of supersonic gas to regulate the pressure-displacement behavior of the mechanism. Compared to traditional flapper–nozzle mechanisms, the new negative-pressure nozzle has a wider operating range and higher linearity.

A mathematical model that takes into account gas compressibility was constructed by integrating traditional flapper–nozzle design methodologies. This model yielded the characteristic curves of the flapper–nozzle mechanism that were instrumental in determining the relationship between the diameters of the throttling hole and the nozzle. The fluid domain model underwent grid generation and grid independence verification. The meshing scheme was determined based on the distribution of flow velocity and the convergence of turbulence kinetic energy. Simulation analyses were conducted on the internal flow field within the nozzle and revealed the existence of shock waves. The formation of these shock waves was attributed to the decrease in flow velocity, which subsequently resulted in an increase in pressure, temperature, and density upon the jet’s entry into the receiving orifice, thereby leading to the generation of compression waves.

The simulation results suggested that to prevent the formation of shock waves, the maximum displacement of the flapper should not surpass 70 μm. The selection of the convergent–divergent nozzle was further examined using orthogonal experimental methods, culminating in the choice of a nozzle with a contraction angle of 30°, a contraction length of 1 mm, an expansion angle of 5°, and an expansion length of 1.5 mm.

The complete negative-pressure flapper–nozzle mechanism underwent simulation, producing the displacement-pressure characteristic curve. The simulation results demonstrated that the mechanism exhibits robust linearity within its operational range. Experimental validation was carried out, and the results for displacement-pressure and flow rate curves were found to be in close agreement with the simulation results, thereby affirming the accuracy of the simulation model. This study lays a solid foundation for future applications of pneumatic high-pressure flapper–nozzle mechanisms.

## Figures and Tables

**Table 1:** Grid division schemes for orifice flow.

Schemes	Number of Orifice Nodes	Number of External Nodes	Grid Growth Rate	Total Grid Number
1	300 × 100	200 × 200	1.5	66,381
2	350 × 150	250 × 250	1.5	106,840
3	450 × 200	300 × 300	1.2	215,465
4	550 × 300	400 × 400	1.1	395,079
5	650 × 350	450 × 450	1.05	524,789
6	750 × 400	500 × 500	1.05	811,908

**Table 2:** Grid division schemes for internal nozzle flow.

Schemes	Number of Orifice Nodes	Number of External Nodes	Grid Growth Rate	Total Grid Number
1	0.1	0.5	1.5	33,970
2	0.05	0.1	1.2	110,981
3	0.02	0.1	1.2	333,599
4	0.01	0.05	1.1	951,110
5	0.005	0.02	1.05	1,843,588
6	0.002	0.01	1.05	3,371,818

**Table 3:** Orthogonal simulation design plan.

Simulation Plan	Contraction Angle	Expansion Angle	Contraction Length (mm)	Expansion Length (mm)
1	15°	0°	0.5	1.5
2	15°	5°	1.5	2.5
3	15°	10°	1	3
4	30°	0°	1.5	3
5	30°	5°	1	1.5
6	30°	10°	0.5	2.5

## Data Availability

Data are contained within the article.

## References

[1] Wang T., Cai M., Kawashima K., Kagawa T. (2005). Model of a nozzle-flapper type pneumatic servo valve and differential pressure control system design. Proceedings of the JFPS International Symposium on Fluid Power.

[2] Burazer J., Skoko D., Bukurov M., Novković Đ., Adžić V., Lečić M., Vorotović G. (2023). Possibility for improving the performance of a differential pneumatic comparator by inclining the measuring nozzle. Measurement.

[3] Murty D.V.S. (2010). Transducers and Instrumentation.

[4] Peter B. (2007). Pneumatic Drives System Design, Modeling and Control.

[5] Kagawa T. (1985). Heat Transfer Effects on the Frequency Response of a Pneumatic Nozzle Flapper. J. Dyn. Sys. Meas. Control.

[6] Wang T., Kawashima K., Kagawa T. (2006). Modelling of a 4-port Nozzle-flapper Type Pneumatic Servo Valve. Systems Modeling and Simulation: Theory and Applications, Asia Simulation Conference.

[7] Gang B., Tinghai C., Yao H., Xiangdong G., Han G. (2011). A nozzle flapper electro-pneumatic proportional pressure valve driven by piezoelectric motor. Proceedings of 2011 International Conference on Fluid Power and Mechatronics.

[8] Razminia A., Baleanu D. (2014). Fractional order models of industrial pneumatic controllers. Abstract and Applied Analysis.

[9] Prsic D., Fragassa C., Nedic N., Pavlovic A. (2019). Describing function of the pneumatic flapper-nozzle valve. Mech. Syst. Signal Process..

[10] Yu SC M., Poh H.J., Tso C.P. (2000). Numerical simulation on the flow structure around the injection nozzles for pneumatic dimensional control systems. J. Fluids Eng..

[11] Yang H., Wang W., Lu K. (2019). Numerical simulations on flow characteristics of a nozzle-flapper servo valve with diamond nozzles. IEEE Access.

[12] Kang S., Kong X., Zhang J., Du R. (2022). Research on Pressure-Flow Characteristics of Pilot Stage in Jet Pipe Servo-Valve. Sensors.

[13] Yang H., Wang W., Lu K. (2019). Cavitation and flow forces in the flapper-nozzle stage of a hydraulic servo-valve manipulated by continuous minijets. Adv. Mech. Eng..

[14] Yang H., Wang W., Lu K., Chen Z. (2019). Cavitation reduction of a flapper-nozzle pilot valve using continuous microjets. Int. J. Heat Mass Transf..

[15] Yang Q., Aung N.Z., Li S. (2015). Confirmation on the effectiveness of rectangle-shaped flapper in reducing cavitation in flapper–nozzle pilot valve. Energy Convers. Manag..

[16] Zhang S., Li S. (2015). Cavity shedding dynamics in a flapper–nozzle pilot stage of an electro-hydraulic servo-valve: Experiments and numerical study. Energy Convers. Manag..

[17] Chu Y., Yuan Z., Chang W. (2020). Research on the dynamic erosion wear characteristics of a nozzle flapper pressure servo valve used in aircraft brake system. Math. Probl. Eng..

[18] Lourier J.M., Huber A., Noll B., Aigner M. (2014). Numerical analysis of indirect combustion noise generation within a subsonic nozzle. AIAA J..

[19] Zhao T., Deng Q., Zhang C., Feng K., Zhou Z., Yuan J. (2020). Orthogonal experimental research on dielectrophoresis polishing (DEPP) of silicon wafer. Micromachines.

[20] Kim Y.H., Lee M., Hwang I.J., Kim Y.J. (2019). Noise reduction of an extinguishing nozzle using the response surface method. Energies.

[21] Hotz C., Haas M., Wachter S., Fleck S., Kolb T. (2023). Experimental investigation on entrainment in two-phase free jets. Fuel.

[22] Colin S., Bonnet A., Caen R. (1996). A New High Supply Pressure Pneumatic Flapper-Nozzle with Linear Behavior. J. Dyn. Syst. Meas. Control.

[23] Aupoix B., Spalart P.R. (2003). Extensions of the Spalart–Allmaras turbulence model to account for wall roughness. Int. J. Heat Fluid Flow.

[24] Avila K., Moxey D., De Lozar A., Avila M., Barkley D., Hof B. (2011). The onset of turbulence in pipe flow. Science.

[25] Su L., Zhang J., Wang C., Zhang Y., Li Z., Song Y., Jin T., Ma Z. (2016). Identifying main factors of capacity fading in lithium ion cells using orthogonal design of experiments. Appl. Energy.

